# Unloading shoes for intermittent claudication: a randomised crossover trial

**DOI:** 10.1186/s12872-017-0716-x

**Published:** 2017-11-28

**Authors:** Garry A. Tew, Ahmed Shalan, Alastair R. Jordan, Liz Cook, Elizabeth S. Coleman, Caroline Fairhurst, Catherine Hewitt, Stephen W. Hutchins, Andrew Thompson

**Affiliations:** 1 Department of Sport, Exercise and Rehabilitation, Northumbria University, Northumberland Road, Newcastle upon Tyne, NE1 8ST UK; 2 General Surgery Department, York Hospital, Wigginton Road, York, YO31 8HE UK; 3 School of Sport, York St John University, Lord Mayor’s Walk, York, YO31 7EX UK; 4 York Trials Unit, Department of Health Sciences, University of York, York, YO10 5DD UK; 5 Department of Occupational Therapy, Prosthetics and Orthotics, Faculty of Health Sciences, Oslo and Akershus University College of Applied Sciences, Pilestredet 44, PB4 St.Olavs plass, N-0130 Oslo, Norway; 6 Directorate of Prosthetics and Orthotics and Podiatry, School of Health Sciences, University of Salford, Frederick Road, Salford, M6 6PU UK

**Keywords:** Peripheral arterial disease, Foot orthoses, Gait, Cross-over studies

## Abstract

**Background:**

The purpose of this study was to assess the functional effects and acceptability of rocker-soled shoes that were designed to relatively “unload” the calf muscles during walking in people with calf claudication due to peripheral arterial disease.

**Methods:**

In this randomised AB/BA crossover trial, participants completed two assessment visits up to two weeks apart. At each visit, participants completed walking tests whilst wearing the unloading shoes or visually-similar control shoes. At the end of the second visit, participants were given either the unloading or control shoes to use in their home environment for 2 weeks, with the instruction to wear them for at least 4 h every day. The primary outcome was 6-min walk distance. We also assessed pain-free walking distance and gait biomechanical variables during usual-pace walking, adverse events, and participants’ opinions about the shoes. Data for continuous outcomes are presented as mean difference between conditions with corresponding 95% confidence interval.

**Results:**

Thirty-four participants (27 males, mean age 68 years, mean ankle-brachial index 0.54) completed both assessment visits. On average, the 6-min walk distance was 11 m greater when participants wore the control shoes (95% CI -5 to 26), whereas mean pain-free walking distance was 7 m greater in the unloading shoes (95% CI -17 to 32). Neither of these differences were statistically significant (*p* = 0.18 and *p* = 0.55, respectively). This was despite the unloading shoes reducing peak ankle plantarflexion moment (mean difference 0.2 Nm/kg, 95% CI 0.0 to 0.3) and peak ankle power generation (mean difference 0.6 W/kg, 95% CI 0.2 to 1.0) during pain-free walking. The survey and interview data was mixed, with no clear differences between the unloading and control shoes.

**Conclusions:**

Shoes with modified soles to relatively unload the calf muscles during walking conferred no substantial acute functional benefit over control shoes.

**Trial registration:**

Clinicaltrials.gov, Trial Registration Number: NCT02505503, First registered 22 July 2015.

**Electronic supplementary material:**

The online version of this article (10.1186/s12872-017-0716-x) contains supplementary material, which is available to authorized users.

## Background

Intermittent claudication, a common symptom of lower-limb peripheral arterial disease (PAD), is defined as leg pain or discomfort in the calf of one or both legs that occurs during walking and is relieved within 10 min of rest. Although intermittent claudication is not directly life threatening, it can markedly reduce quality of life by limiting walking and other activities of daily living [1]. Qualitative research has identified that the intensity of claudication pain experienced during walking is influenced by several factors, such as the surface walked on (e.g., grass versus tarmac), the incline and speed of walking, and the type of shoes worn [2]. Regarding the latter, it is thought that factors such as the pitch of the shoe and the amount of support that a shoe gives to the ankle joint may influence the metabolic demands of the calf musculature during walking, and thus the speed of occurrence and intensity of claudication pain [3]. Therefore, if specific shoes could be designed to relatively “unload” the calf musculature during walking, then they might be a useful adjunct treatment for people with calf claudication.

Empirical evidence on footwear interventions for intermittent claudication is mixed and sparse [3–5]. A recent pilot study indicated that pain-free walking distance was increased, and the intensity of claudication pain reduced, when participants with calf claudication (*n* = 8) walked at their self-selected walking speed in a specially-designed, rocker-soled shoe compared with when walking in an un-adapted control shoe [3]. The rocker sole used was shaped to place the foot in a relatively plantar-flexed position during the stance phase of gait while simultaneously reducing sagittal plane ankle range of motion. Previous testing in healthy adults (*n* = 12) had suggested a calf-unloading effect of these shoes; peak ankle plantarflexion moment during walking being reduced by 25% versus control [6]. The present study sought to further explore the efficacy and acceptability of similar rocker-soled unloading shoes in a larger sample of people with calf claudication. The primary objective was to assess the immediate effect of wearing the shoes on walking distances and gait. We also sought participants’ opinions about the shoes following a 2-week period of use in the home environment.

## Methods

### Study design and setting

YORVIC (York study of unloading shoes for vascular intermittent claudication) was a single-centre randomised AB/BA crossover trial with a 2-week observational follow-up. Participants were recruited from vascular clinics at York Hospital, and all assessments were conducted at York St John University. Following a screening visit, participants completed two assessment visits up to 2 weeks apart. At each assessment visit, participants completed three standardised walking tests whilst wearing either the unloading shoes or visually-similar control shoes, the order of which was randomly assigned. At the end of the second assessment visit, participants were given either the unloading or control shoes to use in their home environment for 2 weeks, with the instruction to wear them for at least 4 h every day. At the end of this period, participants returned the shoes and a completed survey about them. A sub-sample of participants was also interviewed about their experiences of using the shoes. Participants were able to claim up to £15 per visit towards travel expenses. The study was approved by the NRES Committee for Yorkshire & The Humber - Leeds West (Ref: 15/YH/0107), and prospectively registered (ClinicalTrials.gov: NCT02505503). Written informed consent was obtained from participants prior to enrolment.

### Participants

Inclusion criteria were: aged ≥16 years; stable symptoms of intermittent claudication for ≥3 months; resting ankle-brachial index ≤0.9 and/or imaging evidence of PAD; pain-free walking distance <250 m on 6-min walk test with ambulation limited primarily by calf claudication (assessed at screening visit), and; able to read and speak English and provide written informed consent. We excluded people with: absolute contraindications to exercise testing (as defined by the American College of Sports Medicine [7]); critical limb ischemia; lower-limb amputation; co-morbidities that limit walking to a greater extent than intermittent claudication (e.g., severe knee osteoarthritis); ambulation limited by claudication in regions other than the calf; major ankle or foot pathology, and; current or previous (within 6 months) use of shoe inserts, knee or ankle braces or customised shoes prescribed by a health professional.

### Interventions

**Fig. 1:**
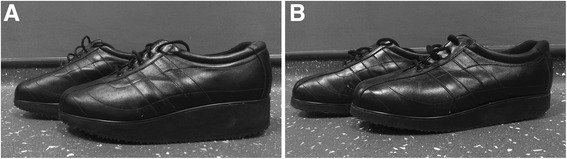
Unloading shoes (**a**) and control shoes (**b**)

The unloading and control shoes were produced and supplied by an established shoe manufacturer (Chaneco; www.chaneco.co.uk). Shoe size was assessed during the screening visit, and shoes were ordered after eligibility had been confirmed. The unloading shoe was a trainer-type shoe with a black leather upper section, laces, and a specially-designed rocker sole (Fig. 1a). The rocker soles, which were manually shaped according to the specifications of the patent that is owned by the University of York (Patent no.: GB2458741B), comprised three circular curves with arc centres that are positioned at the anatomical ankle, hip and knee, respectively (assuming a vertical lower limb), and so forming a posteriorly-placed apex to the rocker shape. This is designed to influence the line of action of the ground reaction force to pass close to the anatomical joint centres and so reduce the moments needed to be generated for ambulation by the muscles acting across those joints in the lower limb. Additionally, it is designed to place the ankle into a relatively plantarflexed position where the ankle plantarflexors use less energy than, for instance, when placed in dorsiflexion. This is because it also increases the lever arm between the Achilles tendon and the ankle joint; so making propulsion, and therefore calf muscle power generation, more efficient. It is also intended to unload the calf muscles by providing a simultaneous reduction in ankle range of motion in relative plantarflexion but still moving with a near-normal trajectory. To facilitate participant blinding, the control shoes were made to be similar in appearance to the unloading shoes (Fig. 1b). These shoes had the same upper section as the unloading shoes, but a different rocker sole. Here, the apex of the sole was anteriorly-placed, which is not designed to place the ankle in relative plantarflexion during stance phase of gait. Participants were allowed to habituate to wearing each pair of shoes for 5 min before commencing the first walking test.

### Assessment procedures and outcome measures

Both assessment visits involved three walking tests that were separated by 20-min periods of seated rest: (i) a 6-min corridor walk test to quantify 6-min walk distance (6MWD) [8], (ii) a usual-pace walk test to measure pain-free walking distance, and (iii) a “figure-of-8” walk test during which gait biomechanical parameters were quantified as described previously [9]. Heart rate (via telemetry: Polar T31 transmitter with Polar FT1 watch, Polar Electro, Oy, Finland), blood pressure (Omron M6 Comfort, Omron Healthcare Europe B.V., Hoofddorp, The Netherlands), and ratings of perceived exertion (Borg 6–20 scale [10]) and leg pain (Borg CR-10 scale [10]) were recorded before and immediately after each test. All participants had a leg pain score of 0 before commencing the next test. For the 6-min walk test, we used a 30-m straight corridor and standardised instructions [11], which included to walk as far as possible within the 6 min. The same course was used for the usual-pace test. The figure-of-8 test was conducted in a gait laboratory. A figure-of-8 was chosen to minimise the potential for fatigue that might have been be caused through participants solely performing all clockwise or all counterclockwise turns. Reflective markers were positioned on anatomical landmarks of the lower extremities using double-sided sticky tape to allow 3D motion analysis [9]. Participants were instructed to walk at their usual pace along a figure-of-8 circuit, without slowing down, for a maximum of 12 min. A force plate (9281EA, Kistler, Germany) positioned in the central straight portion of the course captured kinetic data. The participants were naïve to the force plate, to help ensure a natural walking gait. Infra-red 3D optical motion analysis cameras (Oqus, Qualisys, Sweden) captured kinematic data each time a participant approached and passed over the force plate. Kinetic and sagittal plane kinematic data were exported to Visual 3D motion analysis software (C Motion, Rockville, MD, USA) for processing and analysis. Inverse dynamics were used to determine joint moments and powers. Participants indicated when they experienced the onset of claudication pain and continued walking until pain prevented them walking further. Time-distance variables used to identify gait differences between the two shoe conditions during pain-free walking were walking speed, step length, step cadence, and time in stance phase, swing phase, and double support (% of gait cycle). The potential calf unloading effect of the adapted shoes was also explored using the following variables for the most affected limb: ankle range of motion, peak plantarflexion angle, peak plantarflexion moment (in Nm per kg body mass), and peak plantarflexion power (in W per kg body mass).

After completing the second assessment visit, all participants were given the pair of shoes that they wore during that visit to wear for 2 weeks. During this period, the participants were instructed to wear the allocated shoes as much as possible every day, with a minimum target of 4 h per day [12]. On completing the 2-week period, the participants were asked to return the shoes along with a completed survey about them. In the survey, participants were asked to estimate, on average, how many hours per day they wore the shoes. They also rated the overall level of shoe comfort using an 11-point (0–10) numeric rating scale (with terminal descriptors of ‘extremely uncomfortable’ and ‘extremely comfortable’), and perceived changes in walking ability and physical activity using 5-point (1–5) scales (with terminal descriptors of ‘much worse’ to ‘much better’ and ‘much less physically active’ to ‘much more physically active’, respectively). Finally, participants recorded any benefits, negative aspects, and untoward medical events related to the shoes.

A sub-sample of 12 participants also undertook a telephone-based interview to share their thoughts about the shoes. Purposive sampling was used according to the following criteria: shoe type (unloading and control), age (above and below 65 years), sex, and walking ability (6MWD above and below 350 m). The interviewer sought feedback regarding factors affecting shoe usage, benefits and negative consequences of wearing the shoes, and the design of the shoes. All interviews were audio-recorded, transcribed, and analysed to identify themes.

### Adverse events

We recorded all serious adverse events (regardless of cause), and all non-serious adverse events that were believed to have occurred as a result of performing a study assessment, or from using the study shoes. The latter are subsequently termed ‘adverse device effects’.

### Randomisation, allocation concealment and blinding

The order of testing for each participant (i.e., unloading shoes first then control shoes, or vice versa) was determined using a computer-generated randomisation sequence created by a statistician at York Trials Unit, who was not otherwise involved in the study. Blocked randomisation with a block size of 8 was used to ensure that the overall order of testing was balanced (ratio 1:1). The allocations were blinded (i.e. labelled AB and BA) before being passed to the trial statistician. Once a participant had completed the screening visit, an investigator emailed the trial statistician who assigned the participant to the next available allocation.

Participants were blinded to allocation by using control shoes that were visually-similar to the unloading shoes and by stating in the participant information sheet that the study was investigating two different types of shoes, rather than comparing normal and adapted shoes. Our attempt to blind the outcome assessor was unsuccessful because they were not naïve to the true purpose of the study and therefore could tell which shoe was the unloading shoe when preparing the participant for the gait analysis. However, the use of standardised testing procedures and objective outcomes (e.g., 6MWD) ensured that the risk of detection bias is low. The researcher overseeing data entry and the statistician remained blinded until the analysis was complete.

### Sample size

The primary outcome was 6MWD measured in metres. The cross-over ANOVA square root of the mean squared error for 6MWD was found to be 30 m in a recent trial [13]. A mean difference of 25 m has been suggested as the minimum clinically important difference [14]. Using these values at 90% power and 2-sided 5% significance level in a cross-over design would require 34 participants. Therefore, recruitment stopped once 6MWD had been collected at both assessment visits for 34 participants.

### Statistical analysis

Formal analyses were conducted following the principles of intention-to-treat with participant’s outcomes analysed according to their original, randomised testing order irrespective of the order that they actually received the shoes, where data were available. Analyses were undertaken in Stata v13 using two-sided statistical tests at the 5% significance level. Participant baseline data are summarised descriptively overall and by testing order (AB or BA) both as randomised and as analysed in the primary analysis. No formal statistical comparisons between testing orders were undertaken on baseline data. To allow for a possible period effect, analysis of the 6MWD was via a two-sample t-test to compare the difference between assessment 1 and assessment 2 for the two sequences. Dividing the resultant difference (and corresponding 95% confidence limits) by two gives an estimate of the treatment effect (i.e., A minus B) and 95% CI. Pain-free walking distance at usual pace was analysed in the same manner. Kinetic, kinematic and temporal-spatial measures of gait were taken at each assessment visit and calculated for each participant when pain free, at the onset of pain, and at absolute pain. The difference between the measure as assessed at visit 1 and visit 2 was calculated for each participant at each point in time (pain free, pain onset, and absolute pain). These three differences were modelled using a covariance pattern mixed model, with sequence allocation (AB or BA), time and an allocation-by-time interaction as fixed effects and participant as a random effect. The mean differences (and 95% CI) between the two sequences were extracted for the pain-free, onset of pain, and absolute pain time points, and divided by two to obtain an estimate of the treatment effect A-B. Only the data for pain-free walking is presented in this manuscript; all other gait data will be published elsewhere.

## Results

**Fig. 2:**
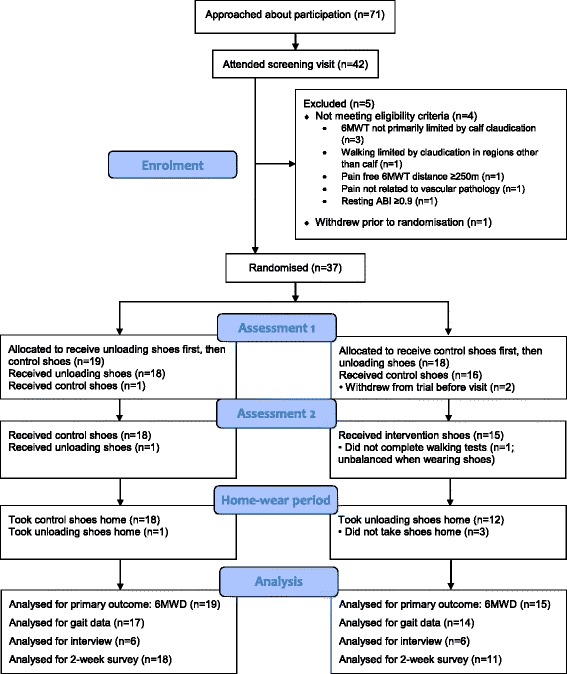
Flow of participants through the trial

Between August 2015 and August 2016, 71 patients were approached to participate in the trial, of whom 42 (59%) were screened and 37 (52%) were randomised (Fig. 2): 18 were allocated to the sequence AB (control, unloading) and 19 to BA (unloading, control). Two participants withdrew from the trial before the first assessment visit, and one participant withdrew during the second assessment visit due to an adverse device effect of feeling unbalanced whilst wearing the shoes; resulting in 34 participants being included in the primary analysis. Characteristics of the 37 randomised participants and the 34 analysed participants are presented in Table 1. The majority of participants (as randomised) were male (*n* = 27, 73%), and the mean age was 67 years (range 31 to 86). All participants had experienced symptoms of intermittent claudication for at least 4 months (median 16 months) at screening, and had a resting ankle-brachial index for the most-affected limb of between 0.25 and 0.89 (mean 0.53, SD 0.14). The mean distance walked during the 6-min walk test at screening was 373 m (SD 100).

**Table 1:** Baseline characteristics of the participants as randomised, and as included in the primary analysis

Characteristic	As randomised			As analysed		
	AB (*n* = 18)	BA (*n* = 19)	Total (*n* = 37)	AB (*n* = 15)	BA (*n* = 19)	Total (*n* = 34)
Age, years, mean (SD)	67.3 (14.9)	66.5 (10.2)	66.9 (12.5)	70.6 (8.8)	66.5 (10.2)	68.3 (9.7)
Gender, male	12 (67)	15 (79)	27 (73)	12 (80)	15 (79)	27 (79)
Ethnic origin, White British	18 (100)	19 (100)	37 (100)	15 (100)	19 (100)	34 (100)
Ankle-brachial index, mean (SD)	0.52 (0.13)	0.54 (0.16)	0.53 (0.14)	0.53 (0.13)	0.54 (0.16)	0.54 (0.14)
Duration of claudication symptoms, months, median (range)	15 (6, 125)	30 (4, 249)	16 (4, 249)	15 (6, 125)	30 (4, 249)	18 (4, 249)
Body mass index, kg/m^2^, mean (SD)	27.8 (4.2)	28.4 (4.8)	28.1 (4.5)	28.8 (3.8)	28.4 (4.8)	28.5 (4.4)
Heart rate, beats/min, mean (SD)	73 (12)	72 (11)	72 (11)	75 (12)	72 (11)	73 (11)
Systolic blood pressure, mmHg, mean (SD)	158 (21)	143 (25)	150 (24)	156 (22)	143 (25)	149 (24)
Diastolic blood pressure, mmHg, mean (SD)	83 (12)	81 (10)	82 (11)	84 (13)	81 (10)	82 (11)
Current smoker	4 (22)	2 (10)	6 (16)	4 (27)	2 (10)	6 (18)
Previous smoker	12 (67)	14 (74)	26 (70)	11 (73)	14 (74)	25 (73)
Never smoked	2 (11)	3 (16)	5 (14)	0 (0)	3 (16)	3 (9)
Diabetes mellitus	4 (22)	5 (26)	9 (24)	4 (27)	5 (26)	9 (27)
Hypertension	14 (78)	13 (68)	27 (73)	12 (80)	13 (68)	25 (74)
Hyperlipidaemia	14 (78)	14 (74)	28 (76)	13 (87)	14 (74)	27 (79)
Chronic obstructive pulmonary disease	1 (6)	2 (11)	3 (8)	0 (0)	2 (11)	2 (6)
Arthritis	2 (11)	2 (11)	4 (11)	1 (7)	2 (11)	3 (9)
History of angina	2 (11)	6 (32)	8 (22)	2 (13)	6 (32)	8 (24)
History of myocardial infarction	0 (0)	6 (32)	6 (16)	0 (0)	6 (32)	6 (18)
History of stroke/transient ischaemic attack	3 (17)	2 (11)	5 (14)	3 (20)	2 (11)	5 (15)
Anti-platelet/Anti-coagulant medication	13 (72)	14 (74)	27 (73)	12 (80)	14 (74)	26 (77)
Lipid-lowering medication	14 (78)	13 (68)	27 (73)	13 (87)	13 (68)	26 (77)
Anti-diabetic medication	3 (17)	4 (21)	7 (19)	3 (20)	4 (21)	7 (21)
Beta-blockers	3 (17)	4 (21)	7 (19)	1 (7)	4 (21)	5 (15)
Other anti-hypertensive medication	13 (72)	14 (74)	27 (73)	11 (73)	14 (74)	25 (74)
6-min walk distance, metres, mean (SD)	372 (92)	367 (106)	369 (98)	382 (95)	367 (106)	373 (100)

Data are number (%) unless otherwise stated. AB, control then unloading; BA, unloading then control

### Effects of the unloading shoes on walking ability and gait

The first assessment visit took place between 3 and 26 days after screening (median 12 days for the AB group and 11 days for the BA group), and the second visit up to 14 days after the first (median 7 days for the AB group and 5 days for the BA group). All participants received their footwear in the allocated order, except for one participant allocated to BA, who was tested in the order AB (control, unloading) by mistake.

The unadjusted mean 6MWD was 381 m (SD 99) for the control shoe condition (*n* = 35) and 372 m (SD 94) for the unloading shoe condition (*n* = 34). The analysis accounting for a possible period effect indicated that the 6MWD was on average 11 m greater when participants wore the control shoes (95% CI -5 to 26; Table 2); however, this was not statistically significant (*p* = 0.18). The unadjusted mean pain-free walking distance during usual-pace walking was 160 m (SD 88) for the control condition (n = 35) and 164 m (SD 132) for the unloading condition (n = 34). On average, participants walked 7 m further before experiencing pain when wearing the unloading shoes (95% CI -17 to 32; Table 2). Again, this difference was not statistically significant (*p* = 0.55).

**Table 2:** Walking distance results

Outcome measure	Treatment sequence	Treatment period		Within-individual difference: Control minus unloading
		1	2	
6-min walk distance, metres	AB			
	Sample size	16	15	15
	Mean (SD)	386 (92)	374 (93)	16 (45)
	BA			
	Sample size	19	19	19
	Mean (SD)	371 (97)	376 (107)	5 (45)
	Treatment effect^a^			
	Sample size	–	–	34
	Mean (95% CI)	–	–	11 (−5 to 26)
	*p*-value	–	–	0.18
Pain-free walking distance during usual-pace walking, metres	AB			
	Sample size	16	15	15
	Mean (SD)	161 (106)	217 (178)	−53 (81)
	BA			
	Sample size	19	19	19
	Mean (SD)	121 (55)	159 (72)	38 (60)
	Treatment effect^a^			
	Sample size	–	–	34
	Mean (95% CI)	–	–	−7 (−32 to 17)
	*p*-value	–	–	0.55

^a^Estimate of the difference between the control and the unloading shoes, accounting for a possible period effect

AB, control then unloading; BA, unloading then control

*CI* confidence interval, *SD* standard deviation

Mean rating of perceived exertion at the end of the 6-min walk test in the first assessment was 12.1 (i.e., “light” to “somewhat hard”; SD 2.0) in the AB group and 12.1 (SD 1.9) in the BA group. In the second assessment, it was 12.4 (SD 1.9) in the AB group and 12.0 (SD 2.6) in the BA group. The mean difference between conditions (control minus unloading) was −0.1 (95% CI -0.7 to 0.6). Mean rating of leg pain at the end of the 6-min walk test in the first assessment was 5.1 (i.e., “strong”; SD 2.5) in the AB group and 5.0 (SD 2.2) in the BA group. In the second assessment, it was 4.6 (SD 2.2) in the AB group and 5.3 (SD 2.1) in the BA group. The mean difference was 0.5 (95% CI -0.1 to 1.1). Heart rate responses were similar for both conditions (data not presented).

Gait variables for pain-free walking are presented in Table 3. The temporal-spatial variables (e.g., walking speed, step length, step cadence) did not differ substantially between conditions. This was also the case for ankle range of motion (mean difference 0.8°; 95% CI -0.5 to 2.2). However, relative to control, the unloading shoes caused a reduction in the peak values of plantarflexion angle (mean difference 2.5°; 95% CI 1.3 to 3.7), ankle plantarflexion moment (mean difference 0.2 Nm/kg, 95% CI 0.0 to 0.3), and ankle power generation (mean difference 0.6 W/kg, 95% CI 0.2 to 1.0).

**Table 3:** Gait variables during pain-free walking

Variable	Control Shoes A	Unloading shoes B	Mean difference^a^ (95% CI)
Walking speed (m/s)	1.16 (0.26)	1.17 (0.26)	−0.01 (−0.03 to 0.01)
Step length (m)	0.63 (0.11)	0.63 (0.11)	−0.01 (−0.02 to 0.01)
Step cadence (steps/min)	110.0 (11.5)	109.7 (12.1)	0.4 (−0.9 to 1.6)
Stance phase (%)	64.2 (2.3)	63.9 (2.3)	0.3 (−0.1 to 0.7)
Swing phase (%)	35.7 (2.4)	36.0 (2.5)	−0.3 (−0.8 to 0.2)
Double support (%)	28.5 (4.5)	28.1 (4.0)	0.3 (−0.3 to 1.0)
Ankle range of motion (°)	24.4 (3.6)	23.6 (3.4)	0.8 (−0.5 to 2.2)
Peak plantarflexion angle (°)	14.8 (3.2)	12.3 (2.9)	2.5 (1.3 to 3.7)
Peak plantarflexion moment (Nm/kg)	1.4 (0.2)	1.2 (0.4)	0.2 (0.0 to 0.3)
Peak ankle power generation (W/kg)	2.3 (0.8)	1.7 (0.8)	0.6 (0.2 to 1.0)

^a^Adjusted estimate of difference for control minus unloading using covariance pattern mixed model approach

Data are mean (SD) unless otherwise stated. Kinetic and kinematic data are for the most affected limb

*CI* confidence interval

### Adverse events

There were four non-serious adverse device effects: three for the unloading shoes and one for the control shoes. The adverse device effects for the unloading shoes were foot and ankle pain (*n* = 1), perceived difficulty in balancing (n = 1), and irritation of a pre-existing bunion (n = 1). For the control shoes, one participant reported experiencing ‘foot discomfort’. There was also one protocol-related, non-serious adverse event. Here, a participant experienced mild bruising to the medial aspect of the knee upon removal of the reflective marker that was used for the gait analysis.

### Survey and interview responses

Thirty-one (91%) of the 34 participants who attended their second assessment visit were given a pair of shoes (13 unloading, 18 control) to use in their home environment for 2 weeks, of whom 29 (12 unloading, 17 control) returned a completed survey at the end of this period. Twelve participants (7 male, 5 female; 6 unloading, 6 control) were also interviewed after the 2-week home-wear period. Survey responses indicated a mean daily wear times of 4.8 h (SD 2.4) for the control shoes (*n* = 17) and 3.8 h (SD 1.8) for the unloading shoes (*n* = 11, one missing data point). The interview data also showed similar shoe usage, with responses ranging 3 to 7 h per day; however, one participant reported that she stopped using the unloading shoes after three days because they had irritated her bunion. Median survey comfort ratings were 8 (interquartile range (IQR) 5–10) and 6 (IQR 3–10) for the control and unloading shoes, respectively. All survey respondents and 11 out of 12 interviewees perceived their walking ability as unchanged or better during the home-wear period. In the control group, six survey respondents (35%) and two interviewees (33%) reported an improvement versus six survey respondents (50%) and three interviewees (50%) in the unloading group. However, fewer people reported that their physical activity had increased during the home-wear period: four survey respondents (24%) and one interviewee (17%) from the control group and three survey respondents (25%) and two interviewees (33%) in the unloading group. From the survey, the most commonly cited barriers to using the shoes were “lack of comfort” (*n* = 3 [18%] vs. *n* = 2 [17%], respectively), “shoe appearance” (*n* = 5 [29%] vs. n = 2 [17%], respectively), and “impractical” (n = 2 [12%] vs. n = 3 [25%], respectively). The interviews also gleaned mixed feedback about the comfort and design of the shoes. Six participants (50%; three from each group) reported the shoes as being comfortable, and that they would be willing to pay between £30 and £60 for them. However, three participants (1 control, 2 unloading) found them to be uncomfortable. Four interviewees in the unloading group commented on the heel being too big, whereas only one person from the control group commented on size, describing the shoes as “bulky”. Recommendations on design features included having a boot style rather than a shoe (n = 3), having Velcro instead of laces (*n* = 1), and having a choice of colours (n = 2). All but one interviewee (from the control group) expressed a willingness to participate in a potential future study where participants would be required to use the shoes over a 6-month period. Example quotes are shown in Additional file 1.

## Discussion

In this study, shoes with specially-designed rocker soles to reduce calf load during walking offered no immediate functional benefit when compared with control shoes in people with calf claudication due to peripheral arterial disease. The gait analysis data indicated that the rocker-soled shoes did indeed unload the calves of claudication patients during usual-pace, pain-free walking; however, this did not translate into improved walking distances during the standardised walking tests. Following the 2-week home-wear period, approximately one third of participants using the control shoes and one half of participants using the unloading shoes reported experiencing improved walking ability when wearing their allocated shoes. There was mixed feedback regarding the acceptability of the shoes; however, 11 out of 12 interviewees reported they would be willing to taking part in a longer-term shoe study, which suggests that patients are interested in footwear as an intervention for claudication.

Our findings contrast that of previous limited research. A smaller study of 21 people with calf claudication showed that commercially-made, rocker-soled unloading shoes acutely increased usual-pace walking ability, with both the total distance walked and the distance at which patients were initially bothered by symptoms being on average 77 m (37%, *p* < 0.01) and 89 m (91%, p < 0.01) further, respectively, compared with a standard shoe condition [5]. The use of different walking assessments prevents a direct comparison with our findings; however, it is important to note that the “bothered distance” is highly subjective (and thus has poor reliability), and that the reported differences were largely explained by one outlier participant who showed improvements of 710 m and 850 m, respectively. A more recent study of 8 people with calf claudication also showed that unloading shoes, which were similar to those used in the current study, improved pain-free walking distance during usual-pace walking by an average of 19 m (39%, *p* < 0.05) relative to an un-adapted control shoe [3]. Again, a direct comparison cannot be made because of different walking test procedures, but also because slightly different intervention and control shoes were used. Nevertheless, this previous study was limited by a small sample size and lack of participant and tester blinding, which may have biased the results. The aforementioned limitations in the evidence base prompted the current investigation.

The current study was appropriately powered and assessed walking ability at both usual and forced walking paces. Despite the unloading shoes causing mean reductions in peak ankle plantarflexion moment and peak ankle power generation of 14% and 26%, respectively, the mean differences in walking distances between conditions were trivial and in varied direction (pain-free walking distance improved, 6MWD worsened; Table 2). It is unclear why the beneficial effects of unloading shoes on ankle biomechanics seen here and elsewhere [6] did not translate to functional benefit. One possibility is that 5 min was not long enough for the participants to habituate to wearing the different shoes. Although we cannot rule this out, our approach was consistent with what others have done previously [3, 5]. Alternatively, it may be that the biomechanical effects were generally too small to influence walking distances or that claudication symptoms are not as strongly influenced by manipulating ankle biomechanics as we originally suspected. Interestingly, the mean reduction in peak ankle plantarflexion moment was smaller than that reported previously in healthy younger adults (14% vs. 25%) [6]. This difference may have been due to the slight alterations that were made for intervention and control shoes in the present study, e.g., the soles of the unloading shoes being made less deep to increase acceptability to participants, and the soles of the control shoes being “filled in” to facilitate participant blinding. However, given that the absolute reduction in peak ankle power generation was of similar magnitude to the difference previously reported between claudication patients and healthy controls (2.437 [SD 0.445] vs. 2.957 [SD 0.686], *p* < 0.01) [15], we are surprised that changes in walking distances were not observed. It is important to remember that there is no such thing as a “biomechanically inert” shoe to use as a placebo, and we are confident that the shoe design features we selected were appropriate for assessing the functional effects of unloading shoes while maintaining methodological rigor. Nevertheless, further research would be useful to determine the magnitude of calf unloading that is needed to observe an improved walking ability in different claudicants, and to see if engineering of the shoe can produce greater biomechanical effects without compromising safety and acceptability.

Interestingly, the variable effects of footwear on the walking ability of claudicants in the literature [3, 5], and the fact that many of participants reported beneficial effects of both shoe types during the 2-week home-wear period, raises the possibility that some patients are more responsive to biomechanical interventions than others. Placebo effects likely explain at least some of the reported benefits by survey respondents and interviewees for both types of shoes. The reported benefits of both shoe types might, however, also be related to other characteristics that were common to both shoes. Both were cushioned, lace-up shoes with flexible leather uppers, which for some participants may have represented a significant improvement over their usual footwear. Unfortunately, the usual footwear was not recorded.

In conclusion, the main finding from this study was that the unloading shoes were relatively ineffective for improving walking ability in people with calf claudication. Although this finding is disappointing, the concept of a shoe reducing claudication pain remains good. The mainstay of current treatment for intermittent claudication, after best medical therapy, is invasive intervention. We have a duty to continue to explore non-invasive options in the management of claudication to compliment/substitute the sporadic funding of supervised exercise programmes [16]. Further preliminary studies are needed to optimise shoe design and confirm clinical efficacy before long-term effectiveness studies are pursued; however, we believe that the feasibility of a longer-term study is supported by our findings.

## Additional file

Additional file 1: Trial interview data. (DOCX 16 kb)

## Abbreviations

6MWD: 6-min walk distance
PAD: peripheral arterial disease
SD: standard deviation
YORVIC: York study of unloading shoes for vascular intermittent claudication
