# Supplementary material for: Unloading shoes for intermittent claudication: a randomised crossover trial
Source: BMC Cardiovasc Disord. 2017 Nov 28;17:283. doi: 10.1186/s12872-017-0716-x (PMC5704386; doi:10.1186/s12872-017-0716-x)
Supplement: Additional file 1: — Trial interview data. (DOCX 16 kb) [file 12872_2017_716_MOESM1_ESM.docx]

**Additional Table 1.** Trial interview data.

| **Theme** | **Example Quotes** | **Interpretation** |
| --- | --- | --- |
| Use of the shoes | *“I upped it to about three or four, then I had ‘em, one day I had ‘em on all day. It just depends on what you’re doing you see.”* (16, control shoes)  *“all the time… so more than four hours”* (10, control shoes)  *“So overall I, I reckon about, each day, if you add them all up, it's about six hours/six and a half hours, something like that.”* (28, unloading shoes)  *“some days it was a bit longer but average three to four hours”* (18, unloading shoes) | In general participants complied with instructions to wear the shoes for a minimum of 4 hours/day. |
| Factors influencing shoe wear - comfort | *“They were uncomfortable; they were less comfortable than the shoes I normally wear, quite a lot more uncomfortable.”* (5, control shoes)  *“I mean the shoes were perfect, perfectly comfortable… they were gentle on me feet and, and, and, and you didn’t notice there was anything in them”* (16, control shoes)  *“I felt that they were maybe a little bit loose and they made the soles of my feet sore and my ankles ache, and last night when I got in the soles of my feet were very sore.”* (1, unloading shoes)  *“Well I, I sort of started wearing ‘em because, well I had to do the trial, but then I found that I liked wearing ‘em because they were comfy and I felt better in ‘em.”* (18, unloading shoes) | Some participants found the shoes to be uncomfortable due to a bad fit. Others found the shoes to be comfortable. There were no clear differences in the responses between conditions. |
| Factors influencing shoe wear - appearance | *“Well for me, I’m only small and, and they were a bit bulky; they were very light but just a bit bulky. I’m only seven stone...”* (24, control shoes)  *“Well, you know, you aren’t gonna win any fashion competitions with them.”* (5, control shoes)  *“They’re quite smart actually, yes, they were quite all right, you know.”* (8, control shoes)  *“…the actual size of the shoes appeared to be large, they would maybe look better, as I said earlier, they maybe wouldn’t look as big.”* (1, unloading shoes)  *“I thought that, that they sort of did look a bit clumsy to wear with skirts, but I always wear trousers so it didn’t make any difference, but with trousers and that, yeah, they were, they were good, I mean; cos you can’t really see ‘em, can yer?... I’m old-fashioned, you know, if you wear a skirt you wear daintier shoes, you know, court shoes and that, not these big clodhoppers.”* (18, unloading shoes)  *“Yeah, I thought, I thought they looked great… I mean I was expecting them to look ugly, and I did warn everybody, I said "I'm bringing me shoes, I'm getting my shoes, you've not got to laugh". And no, everybody said "Well they're just like, like a pair of black lace-up shoes" you know. So the design's good…”* (32, unloading shoes) | Participants in both groups expressed both positive and negative opinions surrounding the appearance of the shoes. There were more negative comments than positive comments.  Some participants thought that the shoes were too bulky, particularly the unloading shoes. |
| Factors influencing shoe wear – practicalities | *“Just the appearance really, and the practicality of them not being, you know, the, you know, this time of year you, everything’s wet through, isn’t it, so they're not suitable for … So that limited the use of them.”* (11, control shoes)  *“totally impractical for work”* (5, control shoes)  *“The lace snapped. (laughs)”* (11, control shoes) | Some participants stated that the shoes were not suitable for some specific activities, e.g., for working at a building site, for driving, or for walking a dog across a muddy field.  A number of participants also reported that the laces had snapped. |
| Impact on walking ability | *“Oh yeah, it did, I mean it really, it’s really helped (…) with my walking and everything. I want to keep the shoes but unfortunately I can’t, they’ve already gone back.”* (10, control shoes)  *“But wearing them shoes I found that I could walk a little bit further than I was doing in me flat shoes”* (16, control shoes)  *“No. I don’t think so, I think it’s just exactly the same.”* (24, control shoes)  *“Slightly worse… Just get the condition thing to arrive quicker wearing the shoes than not.”* (5, control shoes)  *“My legs started to ache when I went out [without the study shoes], you know, sort of; so I, I did miss them”* (18, unloading shoes)  *“Well even when I was walking away from work, when I went out and I walked a, a few times with me wife, and even she said “[name], you can walk longer periods” cos I hadn’t realised at the time “without stopping.” So yeah, it, it, it must have helped.”* (7, unloading shoes)  *“you were a little unbalanced and you had to work harder when wearing them, compared to wearing other shoes... your walking ability certainly didn't get better... No, no. ”* (23, unloading shoes)  *“I haven’t found any difference at all.”* (1, unloading shoes) | Participants in both groups reported unchanged or positive effect on walking ability. Some participants deemed the trial period too short to be able to determine impact.  One participant in the control group reported a negative impact on walking ability, citing that their claudication pain was brought on faster when wearing the shoes. |
| Impact on physical activity | “Yeah, I would say so, yeah. (doing more activity)” (10, control shoes)  *“No, no. I’ve, I’ve just done exactly what I’ve done before.”* (24, control shoes)  *“Yes, I was more active. (And you think that was related to having the shoes?) Well I can’t see any other reason for it. But I mean it, you know, the weather wasn’t any different to what it had been and, you know, I felt like I, I felt like I wanted to go for a walk... I did do a lot more walking with those shoes on.”* (18, unloading shoes)  *“No, no.”* (28, unloading shoes) | Participants in both groups reported unchanged or positive effect on physical activity in general.  No participants reported a detrimental effect on physical activity. |
| Expectations around outcome | *“I don’t know what I was expecting to be quite honest.”* (16, control shoes)  *“Well yeah, because I, I didn’t think a pair of shoes would actually benefit me, but they have, so.”* (10, control shoes)  *“Not, no, not, no, not really.”* (11, control shoes)  *“I don’t really know… I had no expectations at all; I was asked by the hospital if I would take part in a survey and I said yes I would do; so I didn’t really have any expectations. I was hopeful that I wouldn’t have the pain that I, that I have actually had; so I suppose I was very disappointed that I’ve got pain.”* (1, unloading shoes)  *(I guess were you expecting to walk further or...?) “I think I was, I think I was hoping but (...) to be honest. (laughter) But no, that didn't work out.”* (28, unloading shoes) | A number of the interviewees started the home-wear period with unclear expectations as to whether they would experience benefit or not from wearing the shoes.  Opinions were mixed in both groups with some participants reporting a perceived benefit in terms of improved walking ability and some expressing disappointment because their expectations were not met. |
| Willingness to pay for shoes | *“I could, yeah, yeah, cos that, yeah, cos I’ve, I wouldn’t have done if, if, if it was just sort of like walking and say seeing ‘em, but now I’ve had the taste of ‘em, you know, I would, yeah… Well they’re, they’re a £50 shoe, aren’t they?”* (16, control shoes)  *“Well knowing what I know I wouldn’t buy a pair because I know they wouldn’t do me any good.”* (24, control shoes)  *“I would go out and buy them, yes… To get rid of that pain out of my legs, [I’D PAY] anything.”* (7, unloading shoes)  *“I could actually see myself buying some; I wouldn't wear them all the time but certainly if I was going to, to certain places… I pay on average about £60, 50 to £60 for shoes or trainers at the moment. So yeah, I'd be willing to pay that.”* (28, unloading shoes) | There was mixed feedback from both groups about the willingness to pay for a pair of shoes designed to benefit intermittent claudication. |
| Recommendations for design alterations | Would prefer boots and something that looks more *“relaxed”* (5, control shoes)  *“Well the only negative thing I have against them was sort of the laces. I think if they did come onto market they might be better with Velcro fastening. I, I do suffer with blood pressure and when you’re down now and again, you know, lacing them up I did find that when I got up I, I went a bit dizzy; so if it was like a, a Velcro fastener it would be a lot quicker, and other people that maybe have got arthritis in their hands, maybe they wouldn’t be able to do the laces as, you know, the, the Velcro fastening would be easier for people that have got arthritis in their hands and that (…) quicker.”* (18, unloading shoes)  *“it would be nice if, you know, if you could get different colours because if you was wearing ‘em, which I wouldn’t, but if somebody was wearing ‘em with a skirt they might like a sort of more neutral colour so as they didn’t look so; cos black does make things look big and heavy, doesn’t it?”* (18, unloading shoes)  *“probably making them a bit wider for people with sort of foot problems.”* (32, unloading shoes) | Suggestions made by participants to improve design features included Velcro fastening, different fitting for people with foot problems, different colours, and use of a boot style. |
| Willingness to take part in a future trial | *“Yeah, yeah… Well in my case, I can’t talk for other people, but I think they’re fantastic”* (10, control shoes)  “I do, yes, because I found them comfortable to wear and, and, and I, I thought they was very good.” (7, unloading shoes) | 11 of the 12 interviewees reported that they would be willing to take part in a further study with a 6-month home-wear period. |
